# Machine Learning for Prediction of Recurrence in Parasagittal and Parafalcine Meningiomas: Combined Clinical and MRI Texture Features

**DOI:** 10.3390/jpm12040522

**Published:** 2022-03-24

**Authors:** Hsun-Ping Hsieh, Ding-You Wu, Kuo-Chuan Hung, Sher-Wei Lim, Tai-Yuan Chen, Yang Fan-Chiang, Ching-Chung Ko

**Affiliations:** 1 Department of Electrical Engineering, National Cheng Kung University, Tainan 70101, Taiwan; hphsieh@mail.ncku.edu.tw (H.-P.H.); n26090774@gs.ncku.edu.tw (D.-Y.W.); e94081107@gs.ncku.edu.tw (Y.F.-C.); 2 Department of Anesthesiology, Chi Mei Medical Center, Tainan City 71004, Taiwan; ed102605@gmail.com; 3 Department of Hospital and Health Care Administration, College of Recreation and Health Management, Chia Nan University of Pharmacy and Science, Tainan 71710, Taiwan; 4 Department of Neurosurgery, Chi Mei Medical Center, Chiali, Tainan 722, Taiwan; slsw0219@gmail.com; 5 Department of Nursing, Min-Hwei College of Health Care Management, Tainan 73658, Taiwan; 6 Department of Medical Imaging, Chi Mei Medical Center, Tainan 71004, Taiwan; taiyuanc@mail.cjcu.edu.tw; 7 Graduate Institute of Medical Sciences, Chang Jung Christian University, Tainan 71101, Taiwan; 8 Department of Health and Nutrition, Chia Nan University of Pharmacy and Science, Tainan 71710, Taiwan; 9 Institute of Biomedical Sciences, National Sun Yat-Sen University, Kaohsiung 80424, Taiwan

**Keywords:** machine learning, meningioma, parasagittal and parafalcine, recurrence, MRI, texture

## Abstract

A subset of parasagittal and parafalcine (PSPF) meningiomas may show early progression/recurrence (P/R) after surgery. This study applied machine learning using combined clinical and texture features to predict P/R in PSPF meningiomas. A total of 57 consecutive patients with pathologically confirmed (WHO grade I) PSPF meningiomas treated in our institution between January 2007 to January 2019 were included. All included patients had complete preoperative magnetic resonance imaging (MRI) and more than one year MRI follow-up after surgery. Preoperative contrast-enhanced T1WI, T2WI, T1WI, and T2 fluid-attenuated inversion recovery (FLAIR) were analyzed retrospectively. The most significant 12 clinical features (extracted by LightGBM) and 73 texture features (extracted by SVM) were combined in random forest to predict P/R, and personalized radiomic scores were calculated. Thirteen patients (13/57, 22.8%) had P/R after surgery. The radiomic score was a high-risk factor for P/R with hazard ratio of 15.73 (*p* < 0.05) in multivariate hazards analysis. In receiver operating characteristic (ROC) analysis, an AUC of 0.91 with cut-off value of 0.269 was observed in radiomic scores for predicting P/R. Subtotal resection, low apparent diffusion coefficient (ADC) values, and high radiomic scores were associated with shorter progression-free survival (*p* < 0.05). Among different data input, machine learning using combined clinical and texture features showed the best predictive performance, with an accuracy of 91%, precision of 85%, and AUC of 0.88. Machine learning using combined clinical and texture features may have the potential to predict recurrence in PSPF meningiomas.

## 1. Introduction

Meningiomas are the most commonly diagnosed primary intracranial tumors [1], of which those in the parasagittal and parafalcine (PSPF) location account for 30% [2]. Although most meningiomas are classified as benign tumors according to the 2016 WHO classification system [3], some of these tumors may show progression/recurrence (P/R) within the first years after surgery [4,5,6]. Some studies have reported that PSPF meningiomas recur more frequently than other intracranial meningiomas, and the high recurrence rates may be associated with incomplete tumor resection [7,8,9]. Surgical complications of PSPF meningiomas remain high due to the frequent involvement of the superior sagittal sinus and deep cerebral draining veins [10,11], and are shown to occur in 19–35% of patients [12,13,14]. Motor deficits, hemiparesis, gait disturbances, mutism, and seizures may occur due to injury of the primary motor cortex, cingulated cortex, and corpus callosum [14,15]. Although tumor recurrence can be prevented by implementing postoperative adjuvant radiotherapy, most neurological deficits, seizures, and intracranial hypertension associated with adjuvant radiotherapy occur in treating PSPF meningiomas [16,17]. In clinical practice, one of the main challenges in the treatment of PSPF meningiomas is to identify factors associated with P/R. Conventional magnetic resonance imaging (MRI) findings such as bone invasion, subtotal resection, large tumor size, and parasagittal location have been reported to be associated with P/R in meningiomas [5]. However, most findings have been presented in qualitative terms, and inter-reader variations may occur during MRI interpretation.

Texture analysis is a new radiomic method that extracts a large number of mathematically defined features from a medical image by means of machine learning algorithms. The quantitative information provided exceeds that of human visual assessment [18]. Texture analysis has been used as a radiological tool for differential diagnosis, treatment monitoring, and prognosis prediction in different types of tumors [19,20,21]. Although texture-based ML for the evaluation of tumor grades in meningiomas has recently been reported [22,23], models for predicting tumor recurrence in PSPF meningiomas have not yet been reported. The purpose of this study is to investigate the role of ML for preoperative prediction of P/R in PSPF meningiomas, using combined clinical and MRI texture features.

## 2. Materials and Methods

### 2.1. Ethics Statement

This study protocol was reviewed and approved by our Institutional Review Board (no.: 10902-009). The personal information of all included patients was de-identified before data analysis. Signed informed consent was waived because patient data were analyzed retrospectively and did not affect the healthcare of the included patients.

### 2.2. Patient Selection

A total of 57 consecutive patients (22 men and 35 women with median age 56 years) diagnosed with (WHO grade I) PSPF meningiomas and treated in our institution between January 2007 to January 2019 were included. The diagnosis was made by means of brain MRI and pathological confirmation. All included patients had complete preoperative brain MRI, and postoperative brain MRI follow-up for more than one year. Patients diagnosed with neurofibromatosis (*N* = 2) or with history of preoperative intracranial radiotherapy (*N* = 1) were excluded.

### 2.3. Clinical Data

The Simpson grade resections were determined by reviewing preoperative brain MRI and the first postoperative MRI (3–6 months after surgery) by a neuroradiologist (C.C.K.) and a neurosurgeon (S.W.L.). Judgment was made by consensus in equivocal cases. Simpson grade I to III resections are considered gross total resection, and Simpson grade IV–V resections are considered subtotal resection [24]. In our institution, adjuvant radiotherapy was routinely suggested for patients who had undergone subtotal resection. For 18 subtotal resection cases, 12 patients received adjuvant radiotherapy, and 6 patients refused further radiation. Postoperative adjuvant radiotherapy was performed via fractionated stereotactic intensity-modulated radiotherapy (IMRT) (*N* = 6, dose ranging from 55 to 60 Gy, with 30 to 33 fractions) or stereotactic radiosurgery (SRS) (*N* = 6, median dose of 25 Gy, ranging from 18 to 30 Gy; median fraction of 5, ranging from 3 to 5 fractions). The protocols of adjuvant radiotherapy are provided in Supplementary File S1.

Based on the Sindou classification [25], the degree of superior sagittal sinus invasion by PSPF meningiomas was classified into six types: type 1, meningiomas attached to the lateral wall of the superior sagittal sinus; type 2, invasion of lateral recess; type 3, invasion of lateral wall; type 4, invasion of both lateral wall and roof; and types 5 and 6, total superior sagittal sinus occlusion, with the contralateral wall free of tumor in type 5. The meningiomas were also divided into anterior, middle, and posterior locations according to their origin in the falx. The anterior third location extends from the frontal fossa to the coronal suture, the middle third location from the coronal suture to the lambdoid suture, and the posterior third location from the lambdoid suture to the torcula [26]. The apparent diffusion coefficient (ADC) value (b = 1000 s/mm^2^) for each PSPF meningioma was also measured manually by a neuroradiologist (C.C.K. and T.Y.C.), following previously published studies [6,27].

### 2.4. Evaluation of Progression/Recurrence (P/R)

P/R was evaluated by a neuroradiologist (C.C.K., 12 years of radiological experience) and a neurosurgeon (S.W.L., 16 years of neurosurgical experience) by comparing the postoperative brain MRI findings between the 3–6 months and more than one year of follow-up. Both readers were blinded to the clinical data of the included patients. In equivocal cases, agreement was arrived at by consensus. Based on published studies [6,27,28], P/R was defined as regrowth of tumor in Simpson grade I–III resections (gross-total resection). For Simpson grade IV–V resections (subtotal resection), P/R was defined as progressive enlargement of residual tumor, with a threshold of 10% increase in tumor volume in comparison with postoperative brain MRIs. In determining P/R, interobserver reliability with Cohen k coefficient of 0.9 was obtained. For patients who received postoperative adjuvant radiotherapy, P/R was differentiated from post-irradiation pseudoprogression based on progressive tumor enlargement, not transient volume increase [29].

### 2.5. Image Acquisition

Preoperative brain MRI images were acquired using a 1.5-T (*N* = 52) (Siemens Avanto, Siemens Aera, or GE Signa HDxt) or a 3-T (GE Discovery MR750) (*N* = 5) MR scanner, equipped with eight-channel head coils in each machine. The MRI scanning protocols were as follows: axial and sagittal spin echo T1-weighted imaging (T1WI), axial and coronal fast spin-echo T2-weighted imaging (T2WI), axial fluid attenuated inversion recovery (FLAIR), axial diffusion-weighted imaging (DWI) and ADC map, and axial with coronal contrast-enhanced (CE) T1WI. The detailed MR imaging protocols are shown in Supplementary File S2.

### 2.6. Machine Learning and Classification Methods

**Figure 1:**
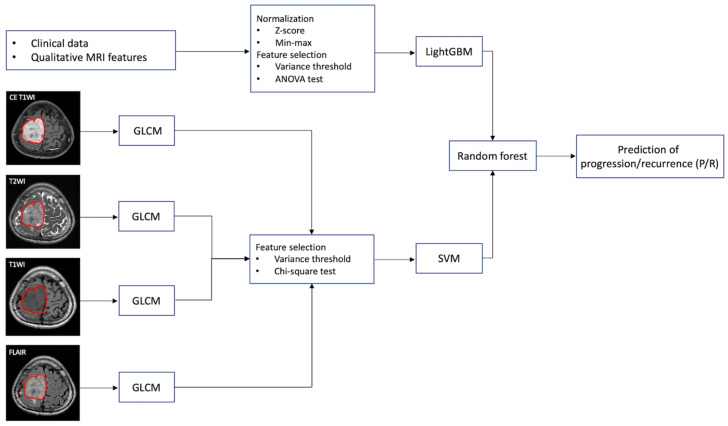
The workflow chart of machine learning analysis for the prediction of progression/recurrence (P/R) in parasagittal and parafalcine (PSPF) meningiomas. Normalization of numerical clinical features are performed first, and feature selection by variance threshold and ANOVA are done. LightGBM is used to perform a prediction based on the 12 most significant clinical features in each patient. For gray-level co-occurrence matrix (GLCM) feature selection, the tumor is first segmented based on contrast enhanced (CE) T1WI, and the region of interest (ROI) of the tumor is then mapped onto the T2WI, T1WI, and FLAIR. On each set of imaging sequences, 84 textural features are extracted, and a total of 336 textural features are collected from each patient. The 73 most significant texture features are selected by variance threshold and chi-square test. Then, support vector machine (SVM) is used to combine these 73 most significant texture features. Finally, the random forest classifier is used to combine the predictive scores from LightGBM and SVM for prediction of P/R in PSPF meningiomas.

T1WI, T2WI, FLAIR, and CE T1WI are known to be associated with histopathology and tumor grades in meningiomas [23,30,31], and the four axial MRI sequences were consistently acquired for all patients and were thus selected for texture analysis. The analysis process is shown in Figure 1. The goal was to predict P/R by combining patients’ clinical and image features. Considering different data characteristics from clinical features and texture features, we applied different machine learning models to check their effectiveness. According to our experimental results, we found that Light Gradient Boosting Machine (LightGBM) and support vector machine (SVM) were good at tackling the clinical features and texture features respectively. To manage the clinical data (shown in Table 1), the LightGBM was used to produce the numerical logits [32]. For the MRI image data, the gray-level co-occurrence matrix (GLCM) was adopted to extract textural features of MRI images [33]. The extracted features were then processed by means of sequential feature selection and SVM to produce the image logits [34,35]. In the final decision stage, the random forest classifier method was adopted to combine the clinical and texture features in differentiating P/R [36,37]. Five-fold cross-validation was applied to test the overall performance of our framework. Based on previously published studies [38,39], we carefully evaluated our framework using cross-validation 15 times to guarantee the validity and effectiveness of the proposed model. By running cross-validation 15 times, we can reduce the variance of the model’s effectiveness, providing a more convincing evaluation. Specifically, each instance in our dataset was used k-1 times for training and once for testing. The present results show the average effectiveness of the proposed methods.

### 2.7. Feature Extraction in Clinical Data

To remove irrelevant clinical features and make accurate predictions, the clinical features that had little variation or were only constant in all patients were filtered. The number of clinical features (Table 1) was reduced from 20 to 16 by conducting variance threshold feature selections [40]. Further analysis of variance (ANOVA) test was applied to each selected feature to measure its relative importance in differentiating P/R. The most significant 12 features were selected and normalized by using Z-score and Min-Max normalization [41]. After preprocessing, the LightGBM was used to perform an initial prediction based on the selected 12 features (Figure 1) [32,42]. LightGBM has shown great success in the medical field [42,43], and is a gradient boosting decision trees (GBDT) algorithm with Gradient-based One-Side Sampling (GOSS) and Exclusive Feature Bundling (EFB) [32]. The process of training LightGBM is iterative. By adding the new model to fix errors made by the model from the previous iteration, gradient boosting can further enhance the overall performance [44]. Furthermore, GOSS performs down sampling for those instances with small gradients, while maintaining the instances with large gradients. Meanwhile, EFB reduces the feature dimensions by bundling exclusive features. Using GOSS and EFB, LightGBM significantly reduces memory consumption and computation cost.

### 2.8. Tumor Segmentation and Texture Feature Extraction

The tumor region was segmented using UNet [45] on all four MRI sequences (Figure 1). Manual correction was performed by an experienced neuroradiologist (C.C.K) in order to prevent under- or over-segmentation. Within the segmented tumor region of interests (ROIs) on CE T1WI, T2WI, T1WI, and FLAIR, textural features were extracted on each sequence using GLCM [33]. GLCM measures the spatial dependency between distinct pixels by calculating how often an intensity x occurs between pixel i and pixel j at certain angles and distances. In this procedure, six texture features, including contrast, dissimilarity, homogeneity, energy, correlation, and angular second moment (ASM), were adopted to calculate the texture properties of GLCM. Detailed mathematical equations of the textures were described below.

$$\mathrm{Contrast}:\;\sum_{i,\;j=0}^{N-1}P\left(i,\;j\right)*{\left(i-j\right)}^{2}\;\mathrm{Dissimilarity}:\;\sum_{i,\;j=0}^{N-1}P\left(i,\;i\right)*\left|\left(i-j\right)\right|\;\mathrm{Homogeneity}:\;\sum_{i,\;j=0}^{N-1}\frac{P\left(i,\;j\right)}{1+{\left(i-j\right)}^{2}}\;\mathrm{Energy}/\mathrm{ASM}:\;\sum_{i,\;j=0}^{N-1}P{\left(i,\;j\right)}^{2}\;\mathrm{Correlation}:\;\sum_{i,\;j=0}^{N-1}P\left(i,\;j\right)\frac{\left(i-\mu \right)\left(j-\mu \right)}{\sigma^{2}}\;\mu :\;\sum_{i,\;j=0}^{N-1}\mathrm{iP}\left(i,\;j\right)\;\sigma^{2}:\;\sum_{i,\;j=0}^{N-1}P\left(i,\;j\right)*{\left(i-\mu \right)}^{2}$$

where *N* is the number of grey levels, μ represents the mean of GLCM, and $\sigma^{2}$ is variance of GLCM [46,47]. Co-occurrence metrics were calculated for two angles of 0 and 45 degrees, and a textural feature was computed from each co-occurrence metric which has 14 features [48]. Therefore, 84 features were obtained for each MRI sequence. Further, feature-level fusion was performed by concatenating multi-modality GLCM features, and a total of 336 texture features were obtained for each case. Variance threshold feature selection was applied to find the relevant GLCM features, and the importance in differentiating P/R was evaluated using the chi-squared test [42]. The most important 73 features were passed to SVM to generate the SVM score for prediction [49]. In this stage, SVM with Gaussian kernel was used as the objective function [35,50]. The SVM score for each patient was calculated using the following equation based on the selected features.

$$\;f\left(x\right)=\overset{N}{\underset{n=1}{\sum }}w_{n}y_{n}G\left(x_{n},x\right)+b\;G\left(x_{n},x\right)=e^{{-\|-x_{n}-x\|}^{2}}$$

where x is the input features, *N* is the length of the support vector, $w_{n}$ is the parameter, and *b* is the bias. $x_{n}$ and $y_{n}$ are the entries of the supporting vector. $G\left(x_{n},x\right)$ is the Gaussian kernel function that indicates the dot product in the predictor space between x and the support vectors [51].

### 2.9. Combination of Clinical and Texture Classifiers

Because random forest classifier is a widely used method based on ensemble learning, it can reduce the variance of the prediction and further improve the stability [44,52,53]. The clinical and texture classifiers were combined by performing decision-level fusion using random forest. The final prediction model in differentiating P/R was established by two predictive results from LightGBM and SVM (Figure 1). The personalized radiomic score is calculated using the following equation [52]:

$$f\left(x\right)=\frac{1}{B}\overset{N}{\underset{b=1}{\sum }}T_{b}\left(x\right)$$

where B is the number of trees and $T_{b}\left(x\right)$ is the output of the tree, and the final prediction is the average result of the individual tree output.

In the random forest model, we can measure the feature importance of using mean decrease in impurity (MDI) [54]. The importance of features in LightGBM and SVM are 0.459 and 0.5409, respectively. Although the weight of features produced by SVM is slightly greater than features from LightGBM, we can still conclude that these features complement each other. That is, combining these two predictive results using random forest will increase the model performance.

### 2.10. Statistical Analysis

For evaluating the clinical and conventional MRI, chi-square test (or Fisher’s exact test) and Mann–Whitney U test were performed respectively, using statistical package SPSS (V.24.0, IBM, Chicago, IL, USA). Univariate and multivariate analyses in Cox hazard regression model were performed to determine independent risk factors of P/R. The area under receiver operating characteristic (ROC) curve (AUC) was calculated to obtain the optimal cut-off values for prediction of P/R. Kaplan–Meier analysis was used to evaluate progression-free survival (PFS), and the log-rank test was used to evaluate significance. For machine learning algorithms, accuracy, precision, recall, and AUC were calculated. A *p*-value < 0.05 was considered statistically significant in statistical analysis.

## 3. Results

### 3.1. Clinical and Imaging Findings

**Figure 2:**
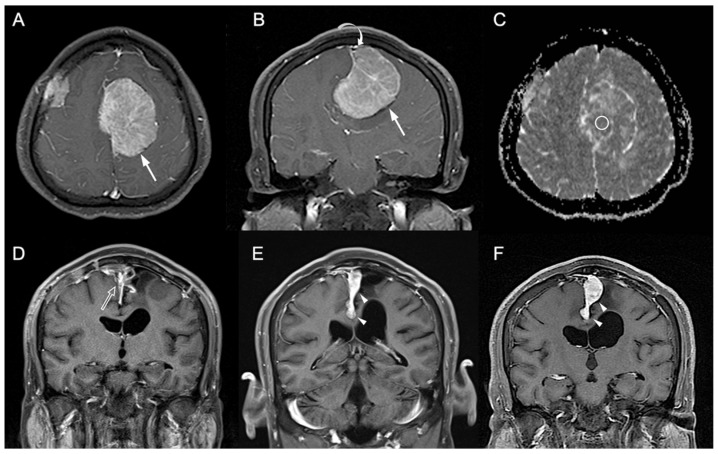
A 41-year-old man with pathologically proven parasagittal meningioma (WHO grade I). (**A**) Axial and (**B**) coronal CE T1WI shows a left parasagittal enhancing tumor mass (white arrow) with invasion into left lateral recess and wall of the superior sagittal sinus (white curved arrow). The radiomic score based on the selected clinical and texture features is 0.623. (**C**) The measured ADC value (circular ROI) is 0.78 × 10^−3^ mm^2^/s (b = 1000 s/mm^2^). (**D**) Subtotal tumor resection is performed to preserve the superior sagittal sinus, and residual tumor (open arrow) is noted in the superior sagittal sinus. (**E**,**F**) Progressive recurrence of tumor (white arrowheads) was observed in 61 months (**E**) and 76 months (**F**) after surgery.

**Figure 3:**
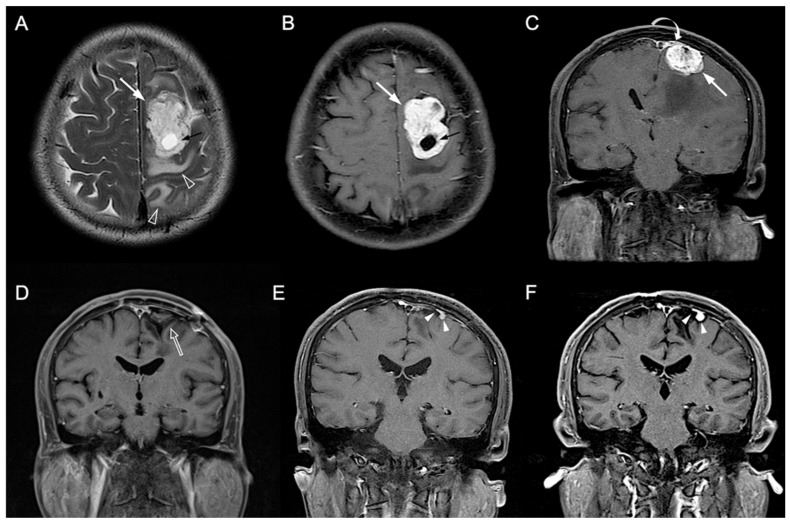
A 54-year-old man with pathologically proven parasagittal meningioma (WHO grade I). (**A**) Axial T2WI and (**B**) CE T1WI shows a left parasagittal enhancing tumor mass (white arrow) with peritumoral edema (open arrowhead) and intratumoral cystic change (black arrow). (**C**) Coronal CE T1WI shows the left parasagittal enhancing mass (white arrow) with invasion of left lateral recess (white curved arrow) of the superior sagittal sinus. The preoperative radiomic score based on the selected clinical and texture features is 0.503. (**D**) Gross total resection is performed, and no residual tumor (open arrow) was detected after surgery. (**E**,**F**) Progressive recurrence of tumor (white arrowheads) was observed in 27 months (**E**) and 47 months (**F**) after surgery.

**Figure 4:**
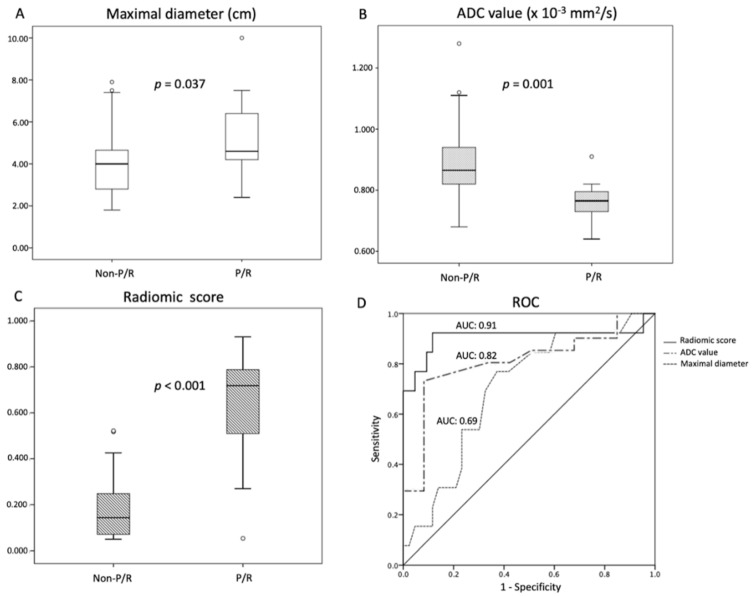
Statistically significant differences (*p* < 0.05) (Mann–Whitney U test) are observed in the box plots of (**A**) maximal diameter, (**B**) ADC value, and (**C**) radiomic scores to differentiate between patients with and without P/R. (**D**) Receiver operating characteristic (ROC) curves of maximal diameter, ADC value, and radiomic scores for the prediction of P/R in PSPF meningiomas, with optimal cutoff values of 4.2 cm, 0.825 × 10^−3^ mm^2^/s, and 0.269, respectively. The AUCs of maximal diameter, ADC value, and radiomic scores in the prediction of P/R are 0.69, 0.82, and 0.91, respectively.

**Figure 5:**
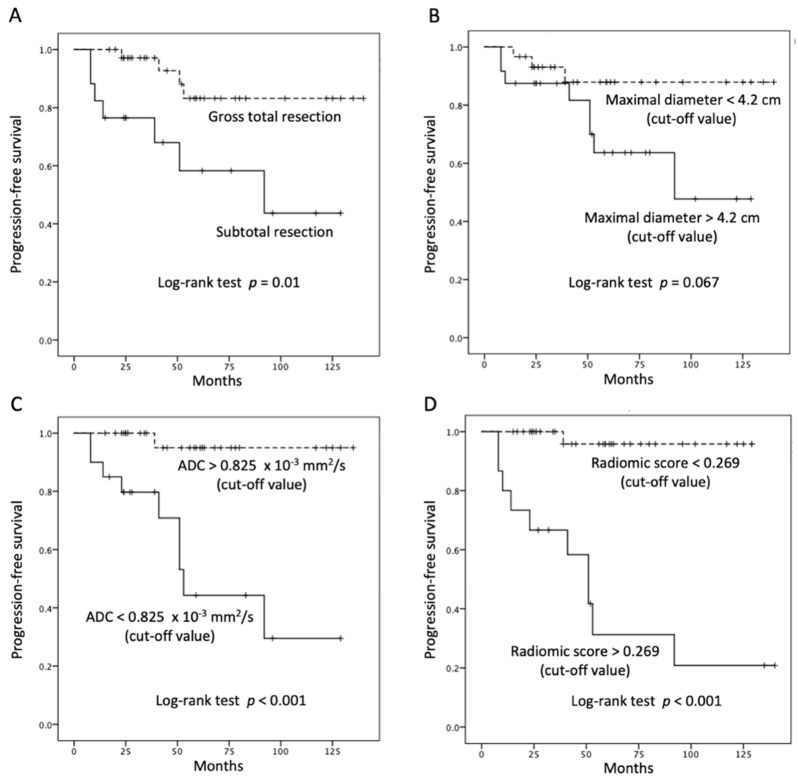
Kaplan–Meier survival curves of (**A**) extent of resection, (**B**) maximal diameter, (**C**) ADC value, and (**D**) radiomic scores for the prediction of P/R in PSPF meningiomas. Extent of resection, ADC value, and radiomic scores show significant differences (*p* < 0.05) (log-rank test) in overall trend of progression-free survival.

Table 1 showed the clinical and imaging findings of the included 57 PSPF meningiomas. Gross-total resection were performed in 39 (39/57, 68.4%) patients, and subtotal resection were performed in 18 patients. Thirteen (13/57, 22.8%) patients were diagnosed with P/R after surgery (Figure 2 and Figure 3). The mean follow-up time was 58.5 months (ranging from 14 to 140 months), and the mean time to P/R was 31.7 months (ranging from 8 to 92 months). Subtotal resection, larger maximal diameter, lower ADC values, and higher radiomic scores were more frequent in the P/R group than in those without P/R (*p* < 0.05). In multivariate analysis, high radiomic score was a high-risk factor for P/R with hazard ratio of 15.73 (*p* < 0.05) (Table 2). For the prediction of P/R, AUCs of 0.91, 0.82, and 0.69 with optimal cut-off values of 0.269, 0.825 × 10^−3^ mm^2^/s, and 4.2 cm were obtained in radiomic score, ADC value, and maximal diameter, respectively (Figure 4). In Kaplan–Meier survival analysis, patients with subtotal resection, low ADC value, and high radiomic scores were found to exhibit shorter PFS (*p* < 0.05) (Figure 5). 

### 3.2. Machine Learning for the Prediction of P/R

**Figure 6:**
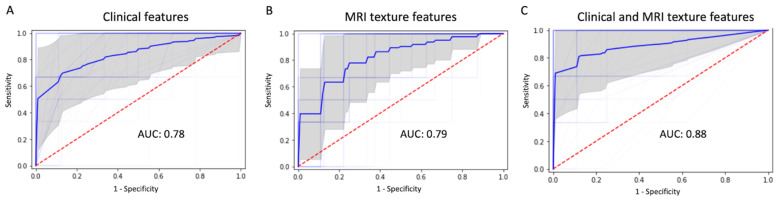
ROC curves (thick blue line: average, thin blue line: five-fold for cross-validation, gray: 95% confidence interval) and AUC values in machine learning model using (**A**) clinical feature, (**B**) MRI texture features, and (**C**) the combination of clinical and texture features for prediction of P/R in PSPF meningiomas.

By using random forest method, the most significant 12 clinical features (extracted by LightGBM) and 73 texture features (extracted by SVM) were combined to predict P/R. The performance of machine learning using clinical data, MRI texture features, and the combination of clinical and texture features, is summarized in Table 3. All metrics were averaged using five-fold cross validation. Compared to using clinical data or MRI only, machine learning using combination of clinical and MRI texture features showed superior prediction performance, with accuracy of 91%, precision of 85%, and AUC of 0.88. (Figure 6).

## 4. Discussion

In the present study, a machine learning model using combined clinical and MRI texture features was constructed for predicting P/R in PSPF meningiomas. The most important 12 clinical features were combined with the 73 textural features extracted from CE T1WI, T2WI, T1WI, and FLAIR to calculate the personalized radiomic score for prediction of P/R. High radiomic score was a significant risk factor for P/R in PSPF meningiomas. Using combined clinical and MRI texture features in machine learning for predicting P/R in PSPF meningiomas was superior to using clinical or MRI data only.

Although most meningiomas are benign tumors, about 13–25% of these tumors may show recurrence within five years after tumor resection [4,8,55]. Radiomic texture analysis is a new method for evaluation of meningioma characteristics. Recently, Zhu et al. [22], Park et al. [56], Yang et al. [57], and Chen et al. [58] used MRI texture-based machine learning to predict the tumor grades and histological subtypes in meningiomas, with accuracy of 76% to 93% and AUCs of 0.81 to 0.92. Morin et al. [30] used radiologic and radiomic features to predict tumor grades and overall survival in meningiomas, with AUCs of 0.75 to 0.78. For prediction of clinical outcomes in meningiomas, Zhang et al. [28] and Ko et al. [6] first applied radiomic features to evaluate tumor recurrence in meningiomas, with accuracy of 90% and AUC of 0.80 respectively. However, most studies used only imaging features without the combined clinical data in machine learning models [6,28]. The application of the radiomic scores is a new concept in precision medicine. By using computer algorithms, X-ray, computed tomography (CT), and MRI imaging features can be transformed into radiomic scores. The process offers useful objective and quantitative values in clinical practice. A personalized radiomic score can be calculated based on selected imaging features in each patient [6,59,60,61,62]. Integration of clinical features into radiomic scores can provide more information in precision medicine [62]. Fan et al. [61] used the radiomic scores to predict radiotherapeutic response in acromegaly, with an AUC of 0.96. Liu et al. [59] reported excellent performance in radiomic scores to evaluate treatment response in rectal cancer, with an AUC of 0.98. Ko et al. [6] first used radiomic scores to predict tumor recurrence in meningiomas, with an AUC of 0.80. Results of these studies suggest that radiomic scores may be a useful tool in predicting tumor recurrence in PSPF meningiomas. Compared with the previously reported studies, the application of machine learning for integration of clinical and imaging features in predicting clinical outcomes in meningiomas has not yet been reported, and no similar studies are available for comparison. In the present results, the superior predictive performance was obtained in CE T1WI among the four MRI sequences, with an AUC of 0.63. After considering all four MRI sequences, the AUC of the predictive model is up to 0.79, which is similar to that of using clinical data (AUC of 0.78). Further, the best performance (AUC of 0.88) can be achieved using a combination of clinical and MRI texture features. Herein, we have introduced this new concept of combining the clinical and imaging features in machine learning for prediction of recurrence in PSPF meningiomas, although the architecture must be validated in further studies with larger sample sizes.

Currently, machine learning using computer-extracted texture features has become a new field in medical imaging. However, the robustness and reproducibility of the radiomic texture features still need to be validated before their clinical practice. The reproducibility of texture features may be associated with image scanners, image reconstruction methods, image preprocessing, and software used to extract imaging features [63]. A recent study showed that 80% of MRI features were repeatable in the test–retest phantom study [64]. Shiri et al. [65] reported up to 74% of MRI texture features of glioblastoma had high reproducibility and robustness. In contrast, another phantom study showed that only one-third (15/45) of features showed excellent robustness and reproducibility across all MRI sequences, and emphasized that care must be taken in the interpretation of clinical studies using non-robust features [66]. However, it appears that operator-dependent bias may be reduced in texture features through fully automatic image segmentation [66], as also shown in the present study. Because LightGBM has shown great success in the medical field [42,43], we chose LightGBM as one strong model for considering clinical data in the present study. Although deep learning-based method such as convolutional neural network (CNN) has shown great success in computer vision, it is hard to collect a large dataset to train CNN models in the present study. In contrast, GLCM is more suitable for our small dataset, and it is less likely to be overfitting [67]. Additionally, GLCM is a powerful model for image classification and has shown great success [68,69]. Thus, we adopt these two techniques as our crucial elements in the present study.

Although low ADC values have been reported to be associated with a higher recurrence rate in PSPF meningiomas [8], manual ROI placement with various methods in ADC measurement may lead to inconsistent results [70]. For meningiomas, the extent of resection is the most significant factor in the tumor recurrence rate [71]. Nanda et al. [71] reported that the overall recurrence rates of WHO grade I meningiomas in Simpson resection grades I, II, III, and IV are 5%, 22%, 31%, and 35%, respectively. Because the tumor may be in close proximity to superior sagittal sinus and large cerebral draining veins, complete tumor (Simpson grade I) resection is often difficult in PSPF meningiomas, especially with invasion of the superior sagittal sinus or adherence to draining veins [13,26]. Although adjuvant radiotherapy improves the tumor control rate in atypical and malignant meningiomas [72], no standard guideline can be adopted regarding adjuvant radiotherapy for benign meningiomas [73]. Whether postoperative adjuvant radiotherapy will be beneficial for benign PSPF meningiomas is still unclear because it has been reported that seizures, motor deficits, and intracranial hypertension caused by adjuvant radiotherapy occur more frequently in PSPF meningiomas [16,17]. Predictive machine learning models, therefore, offers useful preoperative information for determining the treatment strategies. For patients with high risk of P/R, aggressive tumor resection in primary surgery combined with adjuvant radiotherapy and close MRI follow-up should be considered. In contrast, for patients with lower risk of recurrence, the aim of surgery would be to relieve clinical symptoms and to avoid surgical complications. Although adjuvant radiotherapy may affect the independent prediction of P/R in the present study, no statistically significant difference was observed between the P/R and non-P/R groups.

Compared with the previous studies, we first introduced the machine learning model using a combination of clinical and MRI features to evaluate the clinical outcomes in PSPF meningiomas. However, the present study still has several limitations. First, selection bias may exist in this retrospective study, and external validation is lacking. Second, all images were acquired from a single institution. Evaluating the trained predictive model using multi-center data and different MRI protocols to determine the generalizability is necessary in the future. The inconsistent MRI scanner and magnetic field strength may affect the MRI features. Additionally, the extent of tumor resection and adjuvant radiotherapy may affect the independent prediction in texture analysis, although this limitation is inevitable in studies focusing on this topic due to varied treatment protocols in clinical practice [8,27,28,74,75]. Finally, because the sample size of P/R is relatively small, we chose machine learning-based methods (e.g., LightGBM, SVM, and random forest) to make predictions to avoid overfitting. When more cases become available, we believe deep learning-based models such as CNN could be implemented to further boost the predictive performance.

## 5. Conclusions

Machine learning based on preoperative clinical and MRI texture features is a potential tool for the prediction of tumor recurrence in meningiomas. The present study first reported objective and quantitative radiomic scores for prediction of clinical outcomes in PSPF meningiomas. Machine learning-derived radiomic scores may have the potential to offer valuable information in the treatments of meningiomas, including the extent of resection, implementation of adjuvant radiotherapy, and the time interval of MRI follow-up. However, radiomic scores still need to be validated in a larger multicenter study population before clinical applications.

## Figures and Tables

**Table 1:** The clinical data and magnetic resonance imaging (MRI) findings of parasagittal and parafalcine (PSPF) meningiomas with and without progression/recurrence (P/R).

	P/R	Non-P/R	*p* Value
Number of patients	13	44	
Sex			0.053
Male	8 (61.5%)	14 (31.8%)	
Female	5 (38.5%)	30 (68.2%)	
Age (y)	57 (48, 66)	56 (47.5, 64.5)	0.909
Histological subtypes			0.928
Meningothelial (syncytial)	11 (84.6%)	36 (81.8%)	
Transitional (mixed)	1 (7.7%)	3 (6.8%)	
Fibroblastic (fibrous)	1 (7.7%)	5 (11.4%)	
Parasagittal or parafalcine			0.466
Parasagittal	9 (69.2%)	35 (79.5%)	
Parafalcine	4 (30.8%)	9 (20.5%)	
Degree of superior sagittal sinus invasion (Sindou classification)			0.592
None	3 (23.1%)	15 (34.1%)	
Type 1–3	5 (38.5%)	18 (40.9%)	
Type 4–6	5 (38.5%)	11 (25%)	
Tumor location			0.627
Anterior	5 (38.5%)	11 (25%)	
Middle	7 (53.8%)	28 (63.6%)	
Posterior	1 (7.7%)	5 (11.4%)	
Simpson grade resection			0.015 *
Grades I, II, and III (gross-total resection)	5 (38.5%)	34 (77.3%)	
Grade IV and V (subtotal resection)	8 (61.5%)	10 (22.7%)	
Postoperative adjuvant radiotherapy			0.713
Yes	2 (15.4%)	10 (22.7%)	
No	11 (84.6%)	34 (77.3%)	
Peritumoral edema	7 (53.8%)	26 (59.1%)	0.736
Calcification	2 (15.4%)	15 (34.1%)	0.304
Heterogeneous enhancement	4 (30.8%)	20 (45.5%)	0.346
Cystic change or necrosis	3 (23.1%)	8 (18.2%)	0.700
Dural tail sign	7 (53.8%)	21 (47.7%)	0.698
Skull bone invasion	5 (38.5%)	6 (13.6%)	0.102
Reactive hyperostosis	1 (7.7%)	7 (15.9%)	0.667
Multiplicity	2 (15.4%)	1 (2.3%)	0.127
Maximal diameter (cm)	4.6 (3.3, 5.9)	4 (3.1, 4.9)	0.037 *
Tumor volume (cm^3^)	26.5 (13.8, 120)	20.3 (4.3 36.3)	0.224
ADC value (×10^−3^ mm^2^/s)	0.765 (0.729, 0.802)	0.865 (0.799, 0.932)	0.001 *
Radiomic score	0.718 (0.518, 0.918)	0.143 (0.051, 0.236)	<0.001*
Follow-up time (months)	77 (49.5, 104.5)	54 (27, 81)	0.083

Continuous variables were presented as median and interquartile range (IQR). apparent diffusion coefficient (ADC). * Statistical difference (*p* < 0.05).

**Table 2:** Cox proportional hazards analysis for P/R.

	Univariate Analysis		Multivariate Analysis	
	HR (95 % CI) for P/R	*p*	HR (95 % CI) for P/R	*p*
Sex (fraction male)	2.165 (0.659, 7.110)	0.203		
Superior sagittal sinus invasion (Sindou types 4–6)	1.851 (0.563, 6.082)	0.311		
Location (middle)	1.006 (0.293, 3.455)	0.993		
Subtotal resection	4.407 (1.284, 15.126)	0.018 *	2.063 (0.524, 8.125)	0.301
Postoperative adjuvant radiotherapy	0.612 (0.131, 2.855)	0.523		
Peritumoral edema	0.624 (0.190, 2.056)	0.439		
Calcification	0.556 (0.119, 2.590)	0.455		
Heterogeneous enhancement	0.506 (0.134, 1.911)	0.315		
Cystic change or necrosis	1.221 (0.263, 5.676)	0.799		
Dural tail sign	1.964 (0.588, 6.556)	0.273		
Adjacent bone invasion	3.246 (0.941, 11.194)	0.062		
Reactive hyperostosis	0.698 (0.089, 5.468)	0.732		
Multiplicity	3.051 (0.656, 14.186)	0.155		
Maximal diameter > 4.2 cm (cut-off value)	3.223 (0.852, 12.187)	0.085		
Tumor volume (cm^3^)	1.005 (0.995, 1.015)	0.364		
ADC < 0.825 × 10^−3^ mm^2^/s (cut-off value)	17.183 (2.171, 135.986)	0.007 *	4.130 (0.414, 41.161)	0.227
Radiomic score > 0.269 (cut-off value)	28.701 (3.660, 225.068)	0.001 *	15.729 (1.751, 141.292)	0.014 *

* Statistical difference (*p* < 0.05).

**Table 3:** Performance of machine learning using clinical and MRI data for prediction of P/R in PSPF meningiomas.

5-Fold Cross Validation	Data	Accuracy	Precision	Recall	AUC
Average over 15 trials	Clinical data	0.86	0.83	0.67	0.78
	T1WI	0.77	0.53	0.23	0.58
	T2WI	0.68	0.52	0.37	0.57
	T2 FLAIR	0.72	0.43	0.23	0.55
	Contrast enhanced (CE) T1WI	0.77	0.69	0.37	0.63
	Combination of four MRI sequences	0.88	0.83	0.63	0.79
	Combination of clinical and MRI	0.91	0.85	0.83	0.88

## Data Availability

Data available on request due to privacy and ethical restrictions.

## Supplementary Materials

The following supporting information can be downloaded at: https://www.mdpi.com/article/10.3390/jpm12040522/s1, Supplementary Material File S1: Adjuvant radiotherapy protocols; Supplementary Material File S2: MRI protocols.
