# Supplementary material for: Machine Learning for Prediction of Recurrence in Parasagittal and Parafalcine Meningiomas: Combined Clinical and MRI Texture Features
Source: J Pers Med. 2022 Mar 24;12(4):522. doi: 10.3390/jpm12040522 (PMC9032338; doi:10.3390/jpm12040522)
Supplement: Supplementary file 1 [file jpm-12-00522-s001.zip › Supplementary File 2 MRI protocols.pdf]

**MRI Protocols**

The protocols of 1.5T (Siemens, MAGNETOM Avanto) MR imaging were as the following: axial T1-weighted spin-echo (T1WI)(TR/TE, 2000/7 ms; FOV, 22 cm; slice thickness/spacing, 5 mm/6.5 mm; matrix, 320 x 224; voxel size, 0.69 x 0.98 x 5 $mm^3$), T2-weighted imaging (T2WI) (fast spin-echo)(3730/108 ms; FOV, 22 cm; slice thickness/spacing, 5 mm/6.5 mm; matrix, 384 x 261; voxel size, 0.57 x 0.84 x 5 $mm^3$), fluid attenuated inversion recovery (FLAIR) (9000/92 ms; FOV, 22 cm; slice thickness/spacing, 5 mm/6.5 mm; matrix, 256 x 209; voxel size, 0.86 x 1.05 x 5 $mm^3$), and T2-weighted gradient-recalled echo (GRE) (830/26 ms; FOV, 22 cm; slice thickness/spacing, 5 mm/6.5 mm; matrix, 256 x 157; voxel size, 0.86 x 1.4 x 5 $mm^3$). Contrast-enhanced images obtained in axial and coronal T1WI with fat saturation (2000/8 ms; FOV, 22 cm; slice thickness/spacing, 5 mm/6.5 mm; matrix, 320 x 256; voxel size, 0.69 x 0.86 x 5 $mm^3$) were performed after intravenous administration of 0.1 mmol/kg of body weight of gadobutrol (Gadovist; Schering, Berlin, Germany) or gadoterate meglumine (Dotarem; Guerbet, Villepinte, France). The DWI was performed by applying sequentially in the x, y, and z directions with the following parameters: TR/TE, 3800/105 ms; FOV, 23cm; flip angle, 90 degrees, slice thickness/spacing, 5 mm/6.5 mm; matrix, 192 x 192; voxel size, 1.2 x 1.2 x 5 $mm^3$; b = 0, 500, and 1000 $sec/mm^2$. ADC maps were obtained from these imaging data.

The protocols of 1.5T (Siemens, MAGNETOM Aera) MR imaging were as the following: axial T1-weighted spin-echo (T1WI)(TR/TE, 2000/9 ms; FOV, 22 cm; slice thickness/spacing, 5 mm/6.5 mm; matrix, 320 x 224; voxel size, 0.69 x 0.98 x 5 $mm^3$), T2-weighted imaging (T2WI) (fast spin-echo)(3760/99 ms; FOV, 22 cm; slice thickness/spacing, 5 mm/6.5 mm; matrix, 448 x 284; voxel size, 0.49 x 0.77 x 5 $mm^3$), fluid attenuated inversion recovery (FLAIR) (9000/86 ms; FOV, 22 cm; slice thickness/spacing, 5 mm/6.5 mm; matrix, 320 x 218; voxel size,

0.69 x 1.01 x 5 mm$^3$), and T2-weighted gradient-recalled echo (GRE) (830/26 ms; FOV, 22 cm; slice thickness/spacing, 5 mm/6.5 mm; matrix, 256 x 157; voxel size, 0.86 x 1.4 x 5 mm$^3$). Contrast-enhanced images obtained in axial and coronal T1WI with fat saturation (2160/9 ms; FOV, 22 cm; slice thickness/spacing, 5 mm/6.5 mm; matrix, 320 x 224; voxel size, 0.69 x 0.98 x 5 mm$^3$) were performed after intravenous administration of 0.1 mmol/kg of body weight of gadobutrol (Gadovist; Schering, Berlin, Germany) or gadoterate meglumine (Dotarem; Guerbet, Villepinte, France). The DWI was performed by applying sequentially in the x, y, and z directions with the following parameters: TR/TE, 5020/69 ms; FOV, 23cm; flip angle, 90 degrees, slice thickness/spacing, 5 mm/6.5 mm; matrix, 192 x 192; voxel size, 1.2 x 1.2 x 5 mm$^3$; b = 0, 500, and 1000 sec/mm$^2$. ADC maps were obtained from these imaging data.

The protocols of 1.5T (GE Healthcare, Signa HDxt) MR imaging were as following: axial T1WI (TR/TE, 2115/10 ms; FOV, 22 cm; slice thickness/spacing, 5 mm/6.5 mm; matrix, 288 x 224; voxel size, 0.76 x 0.98 x 5 mm$^3$), T2WI (3417/109 ms; FOV, 22 cm; slice thickness/spacing, 5 mm/6.5 mm; matrix, 320 x 256; voxel size, 0.69 x 0.86 x 5 mm$^3$), FLAIR (9002/140 ms; FOV, 22 cm; slice thickness/spacing, 5 mm/6.5 mm; matrix, 256 x 192; voxel size, 0.86 x 01.15 x 5 mm$^3$), and T2-weighted GRE (450/15 ms; FOV, 24 cm; slice thickness/ spacing, 5 mm/6.5 mm; matrix, 288 x 192; voxel size, 0.83 x 1.25 x 5 mm$^3$). Contrast-enhanced axial and coronal T1WI with fat saturation (2115/8 ms; FOV, 22 cm; slice thickness/spacing, 5 mm/6.5 mm; matrix, 256 x 224; voxel size, 0.86 x 0.98 x 5 mm$^3$) with intravenous administration of 0.1 mmol/kg of Gadovist or Dotarem. The DWI was performed by applying sequentially in the x, y, and z directions with the following parameters: TR/TE, 6600/73 ms; flip angle, 90 degrees, slice thickness/spacing, 5 mm/6.5 mm; b = 0 and 1000 sec/mm$^2$. ADC maps were obtained from these imaging data. TR/TE, 6600/72 ms; FOV, 25cm; flip angle, 90 degrees, slice thickness/spacing, 5 mm/6.5 mm;

matrix, 128 x 256; voxel size, 1.95 x 0.98 x 5 $mm^3$; b = 0 and 1000 sec/$mm^2$. ADC maps were obtained from these imaging data.

The protocols of 3T (GE Healthcare, Discovery MR750) MR imaging were as following: axial T1WI (TR/TE, 3400/24 ms; FOV, 22 cm; slice thickness/spacing, 5 mm/6.5 mm; matrix, 352 x 224; voxel size, 0.63 x 0.98 x 5 $mm^3$), T2WI (5200/102 ms; FOV, 22 cm; slice thickness/spacing, 5 mm/6.5 mm; matrix, 384 x 320; voxel size, 0.57 x 0.69 x 5 $mm^3$), FLAIR (10000/95 ms; FOV, 22 cm; slice thickness/spacing, 5 mm/6.5 mm; matrix, 320 x 192; voxel size, 0.69 x 1.15 x 5 $mm^3$), and T2-weighted GRE (567/20 ms; FOV, 22 cm; slice thickness/ spacing, 5 mm/6.5 mm; matrix, 256 x 160; voxel size, 0.86 x 1.38 x 5 $mm^3$). Contrast-enhanced axial and coronal T1WI with fat saturation (2215/ 22 ms; FOV, 22 cm; slice thickness/spacing, 5 mm/6.5 mm; matrix, 320 x 224; voxel size, 0.69 x 0.98 x 5 $mm^3$) after intravenous administration of 0.1 mmol/kg of Gadovist or Dotarem. The DWI was performed by applying sequentially in the x, y, and z directions with the following parameters: TR/TE, 8000/64 ms; FOV, 22 cm; flip angle, 90 degrees, slice thickness/spacing, 5 mm/6.5 mm; matrix, 128 x 160; voxel size, 1.72 x 1.38 x 5 $mm^3$; b = 0, 1000, and 1500 sec/$mm^2$. ADC maps were obtained from these imaging data.
